# Revisiting the pathogenesis of podagra: why does gout target the foot?

**DOI:** 10.1186/1757-1146-4-13

**Published:** 2011-05-13

**Authors:** Edward Roddy

**Affiliations:** 1 Arthritis Research UK Primary Care Centre, Primary Care Sciences, Keele University, Keele, UK

## Abstract

This invited paper provides a summary of a keynote lecture delivered at the 2011 Australasian Podiatry Conference. Gout is the most prevalent inflammatory arthropathy. It displays a striking predilection to affect the first metatarsophalangeal joint as well as joints within the mid-foot and ankle. A number of factors are known to reduce urate solubility and enhance nucleation of monosodium urate crystals including decreased temperature, lower pH and physical shock, all of which may be particularly relevant to crystal deposition in the foot. An association has also been proposed between monosodium urate crystal deposition and osteoarthritis, which also targets the first metatarsophalangeal joint. Cadaveric, clinical and radiographic studies indicate that monosodium urate crystals more readily deposit in osteoarthritic cartilage. Transient intra-articular hyperuricaemia and precipitation of monosodium urate crystals is thought to follow overnight resolution of synovial effusion within the osteoarthritic first metatarsophalangeal joint. The proclivity of gout for the first metatarsophalangeal joint is likely to be multi-factorial in origin, arising from the unique combination of the susceptibility of the joint to osteoarthritis and other determinants of urate solubility and crystal nucleation such as temperature and minor physical trauma which are particularly relevant to the foot.

## Background

Gout is a true crystal deposition disease in which all clinical manifestations are considered to be directly attributable to the presence of monosodium urate (MSU) crystals. It is one of the most prevalent inflammatory arthropathies with a prevalence of approximately 1.4%, and is the most common inflammatory arthropathy in men [1]. Both the prevalence and incidence of gout appear to be rising [2]. The primary risk factor for the development of gout is elevation of serum uric acid (urate) levels, or hyperuricaemia. As uric acid levels rise and exceed the physiological saturation threshold of uric acid in body tissues, formation and deposition of MSU crystals occurs in and around joints.

The propensity of gout for the foot was recognised by the ancient Greeks who referred to it as podagra, literally "foot-grabber" [3]. The name "gout" derives from humoral theory and the Latin word *gutta *or "drop", podagra being thought to arise as a result of the bodily humours falling to the affected body part. Although our current understanding of the pathogenesis of gout is dramatically distant from humoral theory, these observations concerning the intimate relationship between gout and the foot have been reinforced over the centuries and continue today. This review will consider the ways in which gout affects the foot and discuss potential mechanisms underlying this relationship.

## Clinical presentation of gout and involvement of the foot

**Figure 1:**
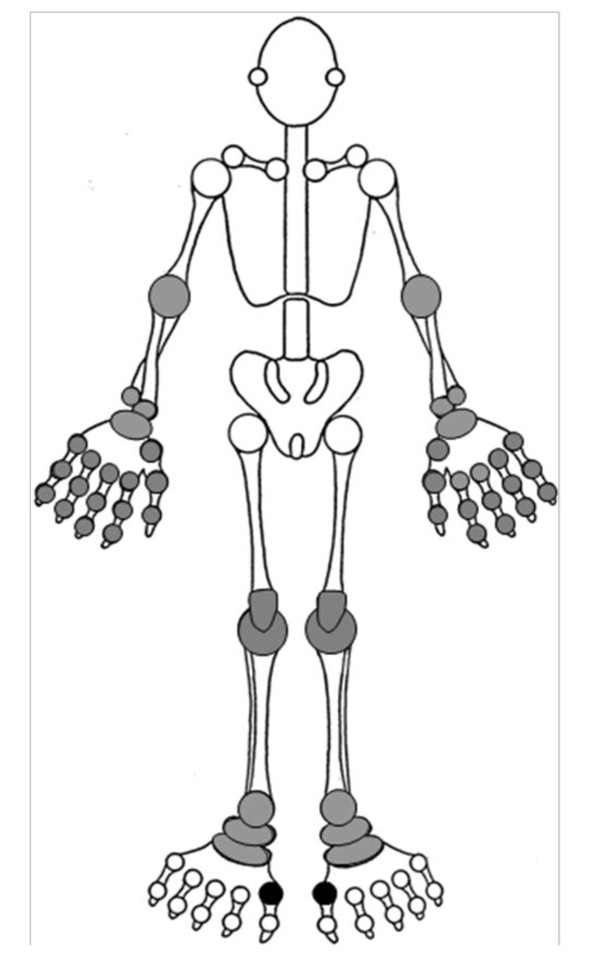
**Distribution of joints typically affected by gout (reproduced with the permission of the author and the Royal College of General Practitioners: Roddy E, Doherty M. Gout**. In: RCGP Guide to MSK Disorders in Primary Care. Ed: Warburton L (in press)).

**Figure 2:**
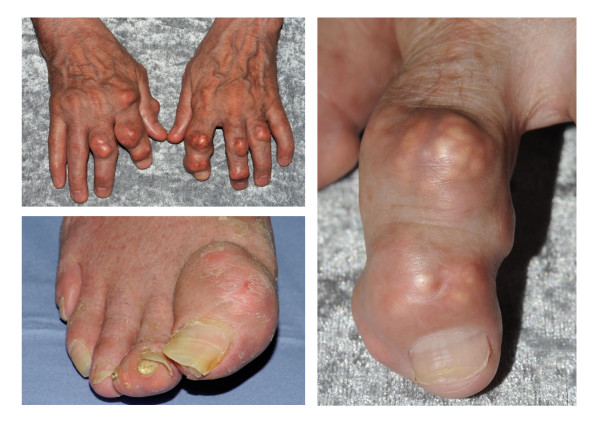
**Tophaceous gout affecting the right great toe and finger interphalangeal joints**. Note the asymmetrical swelling and yellow-white discolouration.

After an often prolonged period of asymptomatic hyperuricaemia, the initial manifestation of gout is usually an acute attack of synovitis affecting a single peripheral joint, most commonly the first metatarsophalangeal joint (MTPJ). Other commonly affected joints include the mid-tarsal joints, ankles, knees, fingers, wrists and elbows (Figure 1). Such attacks are characterised by sudden onset of excruciating joint pain, typically taking less than 24 hours from symptom onset to reach peak intensity, with associated joint swelling, overlying erythema and exquisite tenderness to touch. Although acute gout should be treated rapidly with a non-steroidal anti-inflammatory drug (NSAID) or colchicine, it usually resolves completely over a period of two to three weeks even without treatment. A variable period of time then elapses until the patient experiences a further attack (the "intercritical period"). With time, attacks may increase in severity and frequency, involve different joint sites, and may become oligo- or polyarticular. Eventually, without treatment, the patient may develop chronic tophaceous gout, characterised by chonic pain and stiffness, joint damage and erosive arthropathy, and clinically evident subcutaneous nodular deposits of MSU crystals (tophi) which can occur at the toes, Achilles' tendons, pre-patellar tendons, fingers, olecranon processes, and less commonly, the ears (Figure 2).

Gout displays a striking tendency to affect the foot, in particular the first MTPJ. The initial attack of gout affects the first MTPJ in 56-78% of patients [4-7] and the joint is involved at some point in the course of disease in 59-89% [4,6,8-10]. Fewer studies report the frequency of involvement of other joints. However, mid-foot and ankle involvement occurs in 25-50% and 18-60% of patients respectively [5,8,9]. In contrast, the upper limb is involved in 13-46% [4,6,8,10] and the finger interphalangeal joints in only 6-25% [5,8,9].

Sub-clinical involvement in the foot also appears to be common-place. MSU crystal deposits have been observed in synovial fluid aspirated from first MTPJs that have never been affected by an acute attack of gout [11,12]. Furthermore, a study which examined the first MTPJs of 39 males with gout using high resolution ultrasonography found erosions to be present in 45% of 22 first MTPJs that had never been affected by acute gout [13].

Gout has a number of chronic manifestations which are easily recognisable as such including tophaceous deposits and a characteristic erosive arthropathy. However, it is also associated with a number of other less specific foot problems. Perhaps not surprisingly given the frequency of first MTPJ involvement, hallux valgus is a common finding. In a community-based case-control study, hallux valgus was found in 41% of gout suffers compared to 25% of age- and gender-matched control subjects (odds ratio (OR) 2.10, 95% confidence interval (CI) 1.39 to 3.18, adjusted for body mass index (BMI) and use of diuretics) [14]. Big toe pain occurring on most days for at least a month within the last year was reported by 16% of those with gout compared to 6% of controls (adjusted OR 2.94, 95% CI 1.62 to 5.34). Given the striking predilection of gout for the foot, there has been surprisingly little work examining the influence of gout on foot function, gait and plantar pressure distributions. A recent study compared functional and biomechanical foot characteristics between 25 patients with chronic gout and 25 age- and gender-matched control subjects with no history of gout [15]. Patients with chronic gout were found to have slower walking velocity, reduced step and stride length, reduced peak plantar pressure under the hallux, and higher mid-foot pressure-time integrals compared to controls. The authors postulate that gait pattern is altered in chronic gout in an attempt to off-load the first MTPJ thereby reducing pain. Further studies are necessary to explore these observations in more detail and examine the contribution of chronic pain in the great toe, hallux valgus, obesity and osteoarthritis (OA) to gait patterns in patients with gout.

## Factors influencing crystal deposition

**Figure 3:**
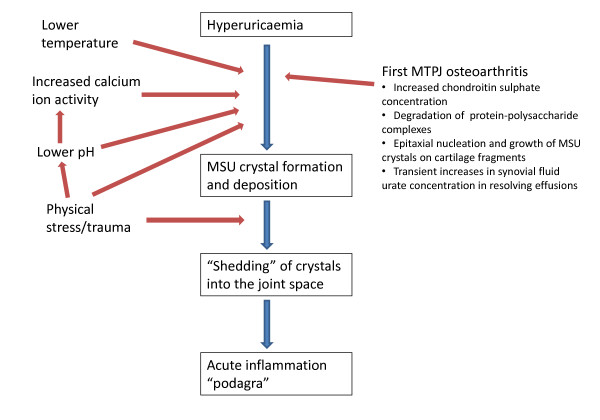
**Processes enhancing MSU crystal formation and deposition at the first MTPJ**.

Gout is one of the best understood inflammatory arthropathies. Clinical features can be easily understood and interpreted in the context of a clearly elucidated pathogenetic process. Specific risk factors such as genetics, dietary factors, co-morbidity and its treatment lead to hyperuricaemia and subsequently MSU crystal formation occurs [16,17]. Crystals are then shed into the joint and activate the inflammatory cascade via the NALP3 inflammasome [18,19]. Hence, any explanation of why gout targets the foot must link these pathological processes to the specific anatomical, functional, and disease characteristics of the foot (Figure 3).

### Temperature

As described above, gout tends to affect distal peripheral joints, not only in the foot but also in the upper limb, with central axial joints such as the shoulders, hips and spine only rarely affected. The solubility of urate decreases with reducing temperature [20,21] enhancing nucleation of MSU crystals, that is, the "birth" of new crystals. Reduced solubility of urate at lower temperatures has therefore been suggested to account for the occurrence of gout at cooler distal joints such as the foot-ankle complex. However, this theory does not account for the preference of gout for the first MTPJ ahead of the great toe interphalangeal (IP) joint or the lesser MTPJs.

### Trauma and pH

A further well-recognised clinical feature of gout is the tendency of an acute attack to be precipitated by physical trauma such as stubbing the toe or following physical activity. Enhanced MSU crystal nucleation has been reported *in vitro *following mechanical agitation of solutions supersaturated with sodium urate [22]. The same authors demonstrated that nucleation is also potentiated by both acidification and addition of calcium ions. Lowering of pH has a direct action on MSU crystal nucleation but also enhances nucleation by increasing calcium ion activity. Whilst their observations concerning mechanical agitation provide evidence that a physical shock can directly lead to MSU crystal nucleation, the authors hypothesised that local trauma indirectly enhances crystal nucleation by lowering synovial pH [22]. Hence, the susceptibility of the foot to physical trauma might also help to explain the predilection of gout for the foot.

### Cartilage damage and osteoarthritis

More recently, the deposition of MSU and calcium pyrophosphate dihydrate (CPPD) crystals in areas of cartilage damage has been described in a cadaveric study which examined 7855 adult human tali from 4007 donors [23]. Crystal deposits, both MSU and CPPD, were an uncommon finding, being present in specimens from only 5% of donors. However, where seen, crystal deposits were usually found within or adjacent to a cartilage lesion. Only 8% of tali with crystal deposits had no gross evidence of cartilage degeneration. Cartilage lesions tended to be located at sites of biomechanical stress such as the articulation of the margin of the trochlea with the tibia or fibula or where apposition with anterior tibial osteophytes was thought to have occurred. In a separate study, the epitaxial nucleation and growth of MSU crystals was observed to occur on fragments of articular cartilage [24]. Thus there appears to be a relationship between cartilage lesions and the anatomical location of MSU crystal deposition.

In support of these observations, clinical and radiographic evidence exists of an association between gout and OA. Several case reports and small case series describe the occurrence of acute attacks of gout and/or tophi at first MTPJs and finger distal interphalangeal (DIP) joints also affected by OA [25-30]. A Polish hospital-based study of 262 patients with gout found an association of gout and radiographic OA at the first MTPJs, tarsal joints and knees [31]. A more recent study of 164 patients with gout recruited from primary care found a very strong association between joints that had previously been the site of an acute attack of gout and evidence of OA on clinical examination (OR 7.94, 95%CI 6.27 to 10.05, adjusted for age, gender, BMI and diuretic use) [8]. Significant associations were seen between acute attacks of gout and the presence of clinical OA at the first MTPJs, mid-foot, knee and finger DIP joints.

## Why are gout and osteoarthritis associated?

The observations outlined above that MSU crystals tend to deposit at sites of cartilage damage and that clinical and radiographic evidence exists of an association between gout and OA lead to the important question of the mechanism by which gout and OA might be associated. There are three possible explanations for this association.

Firstly, does an association exist between the disease states of gout and nodal generalised OA? These two conditions share the common risk factor of obesity [32,33]. In a related study to the primary care study described above [8], generalized nodal OA, defined as the presence of Heberden's or Bouchard's nodes on at least two digits in each hand [34], was no more commonplace in subjects with gout than age-and gender-matched community controls but, as discussed above, hallux valgus and self-reported knee and big toe pain were more frequent in those with gout [14]. Although this case-control study was underpowered, these findings do not suggest that an association exists between the disease states of gout and generalised OA.

The second and third explanations are related and concern the hypothesis that the association of gout and OA occurs at local joint sites and relates to the co-location of MSU crystal deposits and cartilage lesions. Specifically, they question the direction of this association, namely, does the presence of osteoarthritic cartilage predispose to the local formation and deposition of MSU crystals or do MSU crystals themselves initiate and progress cartilage damage? Evidence to support the deposition of MSU crystals in osteoarthritic cartilage rather than MSU crystals leading to cartilage damage arises from two sources. Although the primary care study described above was cross-sectional, making it difficult to infer causality, the strength of the association between involvement of gout and OA at individual joint sites did not increase with longer duration of gout [8]. A further insight into the direction of association between MSU crystal deposition and OA is provided by a recent study which examined the relationship between synovial fluid uric acid levels and the radiographic severity of knee OA [35]. Although synovial fluid uric acid was found to correlate with baseline knee OA severity, it was not associated with change in OA severity over 3 years. These two observations do not suggest that the association between the occurrence of gout and OA at individual joint sites is due to MSU crystal-initiated joint damage. Furthermore, certain properties of the osteoarthritic joint are thought to influence urate solubility and predispose to local MSU crystal disposition [36]. Increased concentrations of chondroitin sulphate and degradation of protein-polysaccharide complexes found within articular cartilage have been shown to reduce urate solubility and lead to the precipitation and growth of MSU crystals [37-39]. However, it is also possible that the association between MSU crystal deposition and OA is bi-directional whereby existing osteoarthritic change predisposes to local formation and deposition of MSU crystals which then initiate further cartilage damage.

## Why does gout target the first metatarsophalangeal joint?

The studies discussed above provide clear evidence of an association between MSU crystal deposition and OA. Whilst further studies are required to definitively answer the questions of direction of association and causality, it appears that MSU crystals more readily deposit in osteoarthritic cartilage and that the presence of OA influences the distribution of joints affected by gout. However, OA cannot solely explain the typical distribution of joints affected by gout, as many joints commonly affected by OA such as the knees, finger IP joints, and hips are less frequently affected by gout than the first MTPJ, and other target joints for gout such as the ankle, wrist and elbow are infrequent sites for primary OA. Is it plausible therefore that the relationship between MSU crystal deposition and OA is of more relevance for the first MTPJ than other joint sites?

The first MTPJ is certainly targeted by OA although foot OA is under-studied in comparison to other commonly affected sites such as the hand and knee. A recent systematic review of population-based epidemiological studies found that the estimated prevalence of radiographic OA at the first MTPJ may be as high as 39% in middle-aged to older adults [40]. Simkin proposed a model to explain the clinical observations that acute attacks of gout are commonly precipitated by physical stress and occur overnight, based upon the co-occurrence of gout and OA at the first MTPJ [41]. In this model, a synovial effusion develops in an osteoarthritic first MTPJ during the day and subsequently resolves when the joint is rested overnight. Synovium is more permeable to water than urate and hence, as the effusion resolves, water leaves the joint more rapidly than urate. This results in a transient increase in the synovial fluid urate concentration which leads to precipitation of MSU crystals if the saturation threshold of urate is exceeded. As discussed above, MSU crystal formation and deposition will be further potentiated in the osteoarthritic first MTPJ by impaired urate solubility and enhanced crystal nucleation arising from factors relating to the anatomical location of the first MTPJ namely lower distal temperature and physical stress [20-22], and those relating to OA namely increased concentrations of chondroitin sulphate, degradation of protein-polysaccharide complexes, and epitaxial MSU crystal nucleation and growth on cartilage fragments [24,37-39] (Figure 3).

## Conclusion

The striking predilection of gout for the first MTPJ appears to be multi-factorial in origin and arises from the unique combination of the susceptibility of the joint to OA and local anatomical considerations of temperature, minor physical trauma and biomechanical stress, leading to ideal conditions for MSU crystal formation and deposition in predisposed hyperuricaemic individuals, manifesting as clinical gout.

## Competing interests

The author declares that they have no competing interests.
